# Ponce De Leon Spring

**Published:** 1875-06

**Authors:** 


*Ponce de Leon Spring.*

I have rented for the season the Ponce de Leon Spring Hotel, situated on a beautiful eminence overlooking the Spring and surrounding country. I am prepared to take boarders who would like to avail themselves of the benefits arising from pure air and the wonderful medicinal virtues of the water of Ponce de Leon Spring.

The street cars run from early morning until late at night, every fifteen minutes, from the centre of the city of Atlanta to the Spring, giving visitors, all the conveniences of city life, and the health-restoring virtues of pure country air, and the far-famed healing quality of Ponce de Leon. MRS. A. M. JOHNSON.

---

[*Letter from Dr. Henry L. Wilson, Atlanta, Georgia.*]

During the year 1870 and the fiist of 1871, I suffered greatly from dyspepsia, and could scarcely digest anything; in fact, I dreaded rather than desired a meal. In a word, I had all those miserable symptoms so universally present in that disease. My energies were broken down to so great an extent that I began to fear my health was entirely gone. I used those remedies usually resorted to, but only received temporary relief. In the Spring of 1871 I was induced to visit a spring near Atlanta, in the vicinity of the Air-Line Railroad, now known as Ponce de Leon. I went at first simply because the distance afforded me a pleasant ride, but finding the water cool, soft and palatable, and the surrounding scenery exceedingly beautiful, grateful and attractive, I continued my visits daily, and in an incredibly short time was rewarded with improvel health. After drinking copiously of the water, continuous eructations followed, leaving me deciedly more comfortable. My appetite increased, my digestion improved, and I soon ate of dishes that I had not touched for a year previous. In a month from the day I first saw Ponce de Leon Spring, I was *entirely* cured of dyspepsia, nor have I experienced the slightest inconvenience since. However, I still drink of this water whenever opportunity presents itself. I also regard it as a superior and harmless diuretic, preferable, perhaps, to anything to be found in the materia medica. I make this statement, hoping that those similarly afflicted may be as fortunate as myself in finding a speedy relief from one of the most wretched diseases human flesh is heir to

Respectfully, HENRY L. WILSON, M.D.

*Atlanta, Ga., April* 4, 1874.

---

[*From the Southern Medical Record.*]

The wonderful and delightful water of the Ponce de Leon Spring has, within our personal knowledge, effected some very remarkable cures, especially affections of the kidneys, of the bladder, and also some remarkable cases of dyspepsia. Nature seems to have gathered here, as in the healing pool of Siloam, some of the rarest and most wonderful remedies for the above-mentioned diseases, which so commonly afflict mankind. This dispensatory of an all-wise Providence opens its treasures to the suffering all the year round, and is a permanent blessing.

Knowing these facts, we deem it our duty to give as much publicity to these waters as possible by means of our journal, in order that all who may be afflicted by such diseases can learn of this beneficent provision of Nature, and, if their physician so advise, partake of its benefits.

We willl give in our next issue the opinion of some who have received the benefits of this delightful and wonderful water. Mrs. Johnson is a lady of the highest character and intelligence, peculiarly fitted to give satisfaction to all who may visit the Springs, either for pleasure or in search of health.

